# MicroRNA-486 as a Biomarker for Early Diagnosis and Recurrence of Non-Small Cell Lung Cancer

**DOI:** 10.1371/journal.pone.0134220

**Published:** 2015-08-03

**Authors:** Wanshuai Li, Yong Wang, Qi Zhang, Lili Tang, Xiaoping Liu, Yunhua Dai, Liang Xiao, Shuguang Huang, Lu Chen, Zhongmin Guo, Jim Lu, Kai Yuan

**Affiliations:** 1 The Department of Cardiothoracic Surgery, No. 2 People's Hospital of Changzhou, 29 Xinglong Xiang, Changzhou, Jiangsu, 213004, China; 2 Changzhou GoPath Diagnostic Laboratory Co. Ltd, 801 Changwuzhong Road, Changzhou, Jiangsu, 213164, China; 3 GoPath Laboratories LLC, 1351 Barclay Blvd, Buffalo Grove, Illinois, 60089, United States of America; The Ohio State University, UNITED STATES

## Abstract

**Background:**

Non-small cell lung cancer (NSCLC) is a leading cause of cancer death worldwide. Early diagnosis is essential for improvements of prognosis and survival of the patients. Currently, there is no effective biomarker available in clinical settings for early detection of lung cancer. Altered expressions in many cancer types including NSCLC and stable existence in plasma make microRNAs (miRNAs) a group of potentially useful biomarkers for clinical assessments of patients with NSCLC.

**Objectives:**

To evaluate the potential values of miRNAs as blood-based biomarkers for early diagnosis and prognosis in NSCLC patients.

**Methods:**

Peripheral blood samples from healthy volunteers and early-staged NSCLC patients before and after surgery were collected, and plasma was separated. Expression of ten miRNAs in the plasma and tumor sections of the patients was detected by quantitative real-time polymerase chain reaction.

**Results:**

MiRNA (miR)-486 and miR-150 were found to significantly distinguish lung cancer patients from healthy volunteers. Area under curve of miR-486 and miR-150 were 0.926 (sensitivity, 0.909; specificity, 0.818) and 0.752 (sensitivity, 0.818; specificity, 0.818), respectively. In response to therapy, patients with down-regulated miR-486 expression showed prolonged recurrence-free survival than those with un-reduced miR-486 expression (median, unreached vs. 19 months; hazard ratio, 0.1053; 95% confidence interval, 0.01045 to 1.060; *P*=0.056).

**Conclusions:**

The results suggest that miR-486 and miR-150 could be potential blood-based biomarkers for early diagnosis of NSCLC. Monitoring change of miR-486 expression in plasma might be an effective and non-invasive method for recurrence prediction of early-staged NSCLC patients.

## Introduction

Non-small cell lung cancer (NSCLC) accounts for approximately 80% of lung cancer, which is a leading cause of cancer deaths worldwide [1]. Over the last few decades, advances in surgical techniques and strategies of chemoradiotherapy and target therapy have significantly improved survival of patients with lung cancer [2, 3]. Despite improvements of clinical approaches in management of lung cancer patients, the survival of patients with locally advanced non-small cell lung cancer (NSCLC) remains poor, with a 5-year overall survival (OS) of 15% for stage III NSCLC patients treated with concurrent chemoradiotherapy [3, 4]. It is estimated that more than 200,000 new lung cancer cases and around 150,000 deaths associated with lung cancer occurs annually in the United States [1]. Early diagnosis represents one of the most effective strategies in improving survival and prognosis of lung cancer patients [2]. However, there is no clinical biomarker in use today for early diagnosis and prediction of prognosis for patients with lung cancer.

MicroRNAs (miRNAs) are single-stranded RNA species that constitute a class of noncoding RNAs, and are emerging as key regulators of gene expression [5]. Many studies have shown that miRNAs exhibit altered expression in various cancers and may serve as important prognostic biomarker of cancers [6–8]. Recent findings that human plasma contains stably expressed microRNA have revealed a great potential of blood-based miRNA signature as facile biomarkers for clinical assessments of human cancers [8–10]. For example, circulating miR-21 was significantly higher in NSCLC patients than that in normal controls [9, 11]. Serum levels of miR-486, miR-30d, miR-1 and miR-499 were significantly associated with overall survival in NSCLC patients [8]. Serum miR-210 was significantly up-regulated in NSCLC patients, compared to healthy control subjects [12].

In this study, we screened the literature and selected ten miRNAs related to NSCLC, including miR-126 [13], miR-150 [14], miR-155, miR-205 [11, 15], miR-21 [9, 11], miR-210 [12], miR-26b [10], miR-34a [7], miR-451 [16] and miR-486 [8, 9], to compare the miRNA levels between healthy volunteers and NSCLC patients.

## Materials and Methods

### Patients

This study retrospectively enrolled early stage patients (before stage IIIa) who had previously undergone lung resections for primary NSCLC between November 2012 and February 2014. The patients were identified from biorepository database of surgical pathology from the Department of Thoracic Surgery, No. 2 People's Hospital of Changzhou, Changzhou, Jiangsu, China. There were no other inclusion or exclusion criteria for this study. Tumors were staged according to the tumor-node-metastasis (TNM) staging system of the American Joint Committee on Cancer [17]. Control subjects were recruited from individuals who sought a routine health check-up at the Physical Health Examination Centre of No. 2 People's Hospital of Changzhou and had not been previously diagnosed with cancer. A 5-ml peripheral venous blood sample was drawn from healthy volunteers and NSCLC patients 2 days before and 7–10 days after surgery, and collected in an anticoagulant tube with ethylene diamine tetraacetic acid (EDTA). The whole blood samples were then centrifuged at 1000 g for 30 min at 4°C in a Sigma 3K15 centrifuge (SIGMA Laborzentrifugen, Osterode am Harz, Germany). Plasma was immediately collected, frozen and stored at -80°C for further analyses. Repeated defrosting was avoided during storage to ensure the quality of the samples. In addition, matched formalin-fixed, paraffin-embedded (FFPE) tissues of the 11 NSCLC patients were collected for following analyses. This study was approved by the Ethics Committee of No. 2 People's Hospital of Changzhou and written informed consent was obtained from all participants of the study.

### RNA extraction and quantitative real-time polymerase chain reaction (qRT-PCR)

RNA extraction was performed as described [12] with slight modifications. Total RNA, including miRNAs, was extracted and purified from 200 μl of plasma or 4 slides of FFPE tumor tissue using the QIAGEN miRNeasy Mini Kit or QIAamp DNA FFPE Tissue Kit (QIAGEN, Hilden, Germany) according to the protocols from the manufacturer. The purity and concentration of RNA were determined using a dual-beam ultraviolet spectrophotometer (Eppendorf, Hamburg, Germany). For quantitative detection of miRNA by RT-PCR [18], purified plasma miRNA was converted to cDNA by reverse transcription reactions using TaqMan MicroRNA Reverse Transcription Kit (Applied Biosystems, Inc., Grand Island, NY, USA) and miRNA-specific stem-loop primers were supplied by the TaqMan MicroRNA Assays (Applied Biosystems, Inc., Grand Island, NY, USA). A total of 10 miRNAs which have been reported to show altered expressions in lung cancer was selected for analysis and their sequences are shown in S1 Table. The RT reactions was mixed according to the manufacturer’s protocol and performed in an Applied Biosystems 9700 PCR instrument using the following conditions: 16°C for 30 min, 42°C for 30 min, 85°C for 5 min, hold at 4°C. Expression levels of the selected miRNAs were tested using quantitative real-time PCR analysis in an ABI 7900HT fast real-time PCR system (Applied Biosystems, Inc., Grand Island, NY, USA). Real-time PCR reactions were performed in a 5 ul reaction mixture containing 2.5 ul TaqMan Universal PCR Master Mix II (Applied Biosystems, Inc.), 0.25 ul miRNA-specific primer/probe mix (Applied Biosystems, Inc., Grand Island, NY, USA), and 2.25 ul diluted RT cDNA template using following cycling parameters: 95°C for 10 min, followed by 40 cycles of 95°C for 15 s and 60°C for 1 min. Reactions were performed in triplicate. Real-time PCR data were collected by SDS 2.2 software (Applied Biosystems, Inc., Grand Island, NY, USA) and relative levels of the tested miRNA in plasma and tissue specimens were calculated using C. elegans synthetic miR-39 and RNU44 as normalization controls, respectively [18–20]. Sequences of both normalization control primers are shown in S1 Table. The cycle threshold (CT), which was defined as the number of PCR cycles required for the fluorescent signal to be higher than a threshold indicating baseline variability, was recorded. Relative gene expression levels of tested samples were represented by 2^-ΔΔCT^, where ΔΔCT = CT_miRNAs_-CT_normalization control_.

### Statistical analyses

Statistical analyses were undertaken using GraphPad Prism version 5.0 (GraphPad Software, San Diego, CA, USA) and the SPSS statistical package, version 16.0 (SPSS Inc., Chicago, IL, USA) for Windows. Data are expressed as the mean±SD. Mann Whitney test was used to analyze the differences in miRNA plasma concentrations between NSCLC patients and healthy volunteers. Receiver operating characteristics (ROC) analysis was undertaken to determine the ability of the plasma miRNA levels to discriminate between NSCLC patients and healthy volunteers. The association between tissue and plasma miRNA levels was analyzed using the Spearman’s correlation coefficient. Log-rank (Mantel-Cox) Test was used to evaluate the association between recurrence-free survival (RFS) and miRNA expression changes before and after surgery. A *P*-value<0.05 was considered statistically significant.

## Results

### MiR-486 and miR-150 were significantly increased in plasma of NSCLC patients

To identify potential blood-based miRNA biomarkers for NSCLC patients, we quantitatively analyzed expression levels of 10 candidate miRNAs in plasma from 11 NSCLC patients and 11 normal individual controls. Clinical and histopathological characteristics of NSCLC patients and control subjects are listed in Table 1. No statistical significant difference was observed in age and gender between patients and control individuals.

**Table 1:** Participants’ characteristics.

Characteristic	Healthy (n = 11)	Lung cancer (n = 11)	*P*
Age, years			0.6932*
Median	55	59	
Range	35–79	41–72	
Sex			1.0000†
Male	6	7	
Female	5	4	

^†^Fisher’s exact test;

*Mann Whitney test.

**Fig 1:**
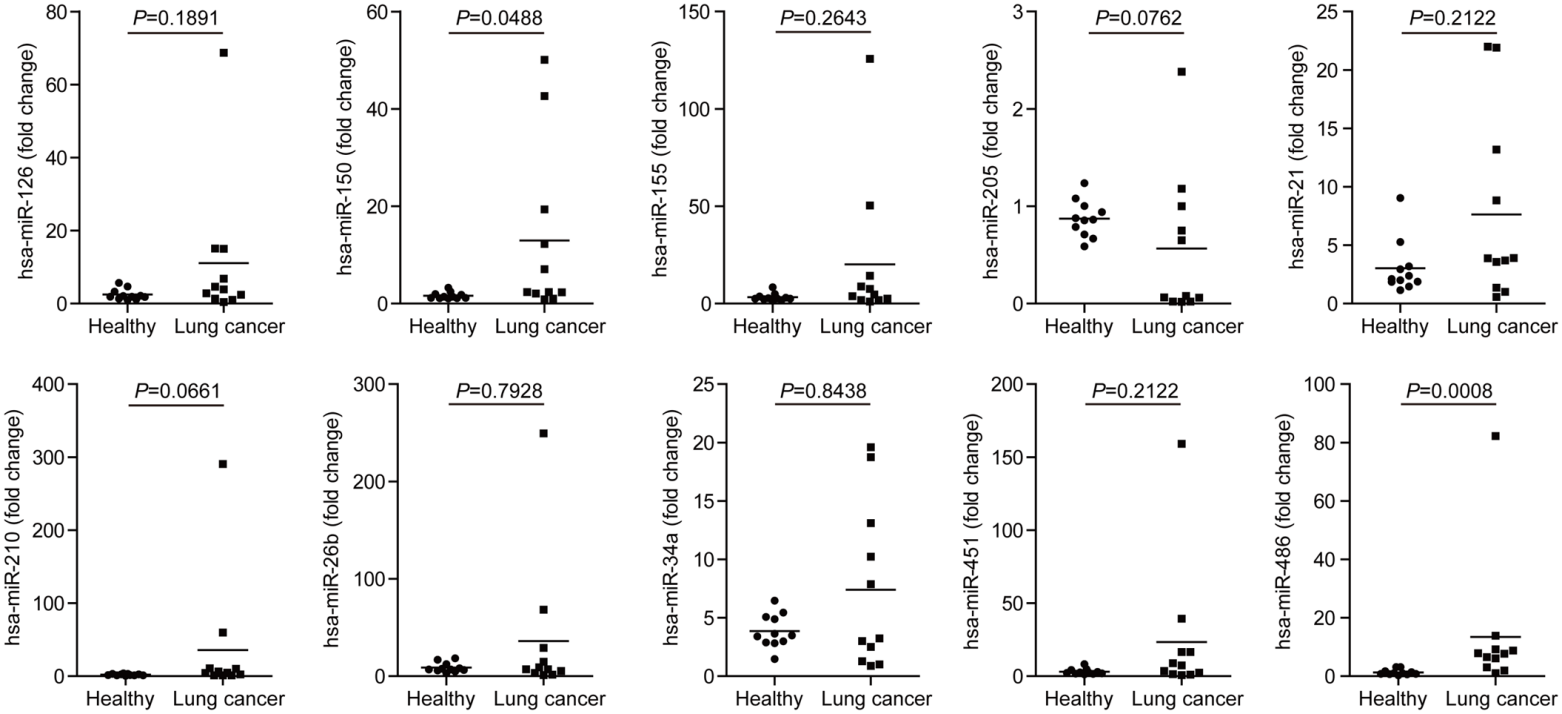
Plasma levels of ten miRNAs. Plasma levels of miR-126, miR-150, miR-155, miR-205, miR-21, miR-210, miR-26b, miR-34a, miR-451 and miR-486 in 11 healthy volunteers (Healthy) and 11 NSCLC patients were analyzed using qRT-PCR and normalized with cel-miR-39. Solid dots showed relative expression of miRNAs in each subject; transverse lines indicated means of miRNAs in each group.

Quantitative analysis of 10 candidate miRNAs consisting of miR-126, miR-150, miR-155, miR-205, miR-21, miR-210, miR-26b, miR-34a, miR-451 and miR-486 in plasma by real-time PCR revealed significantly higher levels of miR-486 (*P* = 0.008) and miR-150 (*P* = 0.0488) in plasma samples from NSCLC patients than those from normal control subjects (Table 2 and Fig 1). No significant differences were observed in plasma levels of the remaining miRNAs between NSCLC patients and normal individuals. Levels of the tested miRNAs in individual plasma sample are shown in Fig 1 and average expression levels of tested miRNAs in plasma of NSCLC and normal control subjects are summarized in Table 1.

**Table 2:** Plasma miRNA level changes before and after surgery.

Case No.	Plasma Levels of miR-150			Plasma Levels of miR-486			Recurrence
	*Before Surgery	*After Surgery	Fold Change	*Before Surgery	*After Surgery	Fold Change	
101	0.512	1.449	2.831	0.421	0.377	0.895	R
102	1.222	1.51	1.236	3.87	1.303	0.337	NR
105	1.07	0.918	0.858	0.813	1.603	1.973	R
118	6.288	3.504	0.557	3.695	2.776	0.751	NR
120	1.181	1.365	1.156	3.323	2.378	0.716	NR
122	0.448	0.939	2.096	1.283	2.322	1.81	NR
127	1.207	0.778	0.645	2.578	0.392	0.152	NR
134	3.615	0.99	0.274	3.229	3.7	1.146	NR
148	9.919	11.103	1.119	2.765	6.622	2.395	NR
152	25.63	6.217	0.243	5.828	7.541	1.294	R
160	21.84	3.077	0.141	34.61	1.939	0.056	NR

*Relative expression;

R, recurrence; NR, no recurrence.

**Fig 2:**
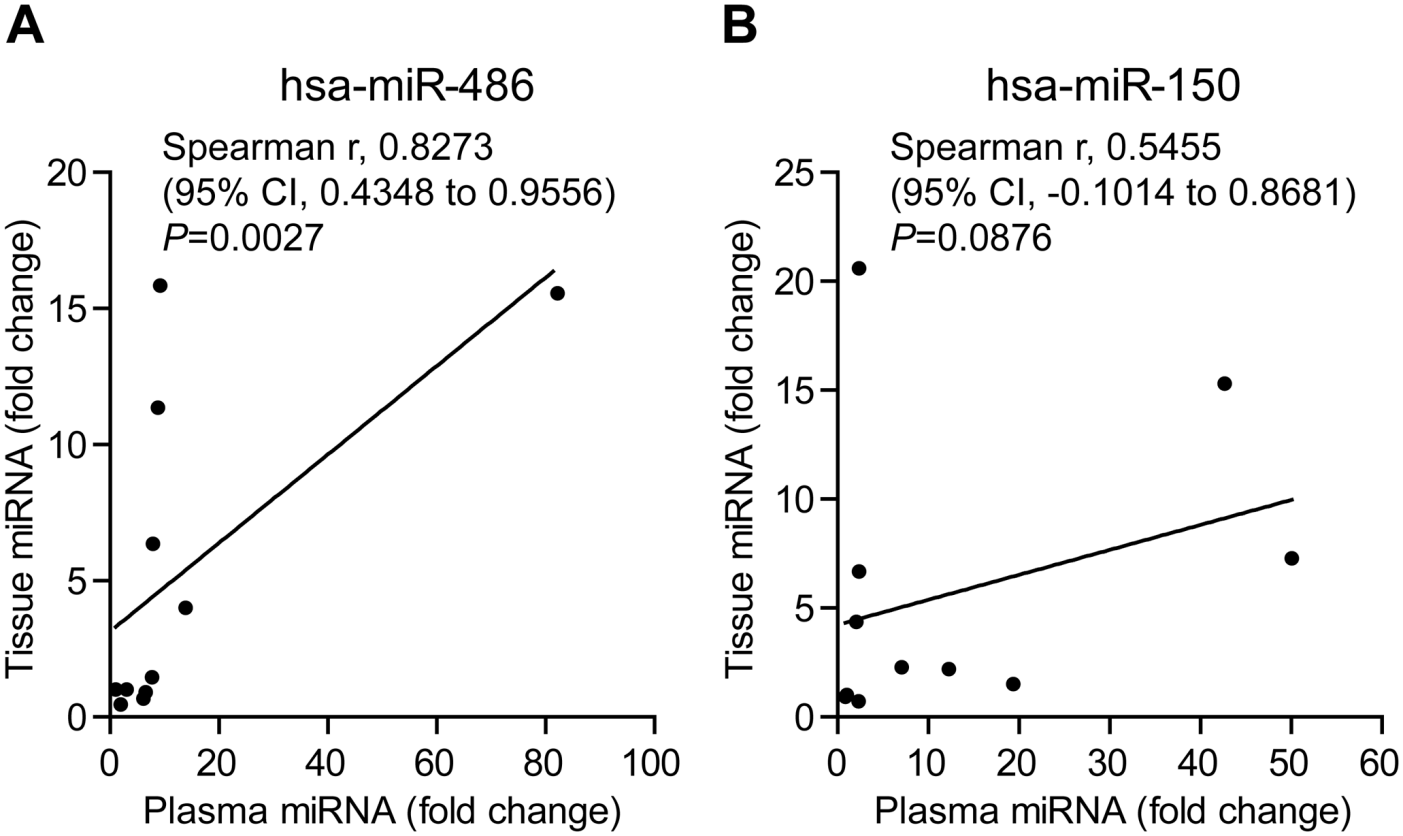
Correlation of plasma levels and tissue expressions of miR-486 and miR-150. Correlation analyses for plasma and tissue levels of miR-486 and miR-150 was performed with GraphPad Prism 5.0 software. (A) Spearman’s correlation coefficient analysis of plasma and tissue miR-486 levels. (B) Spearman’s correlation coefficient analysis of plasma and tissue miR-150 levels.

To determine if higher levels of miR-486 and miR-150 in plasma are associated with their increased expressions in lung cancer tissues, we analyzed correlations of their expression levels between plasma and cancer tissues. Spearman’s correlation coefficient indicated that miR-486 levels in plasma was highly correlated with its expression level in tissue (Spearman r, 0.8273; 95% confidence interval (CI), 0.4348 to 0.9556; *P* = 0. 0.0027), while no significant correlation (Spearman r, 0.5455; 95% CI, -0.1014 to 0.8681; *P* = 0.0876) between plasma and tissue levels for miR-150 was observed (Fig 2). This finding suggests that altered expression of miR-486 in plasma is cancer specific.

### MiR-486 and miR-150 are potential blood-based biomarkers for detection of NSCLC

**Fig 3:**
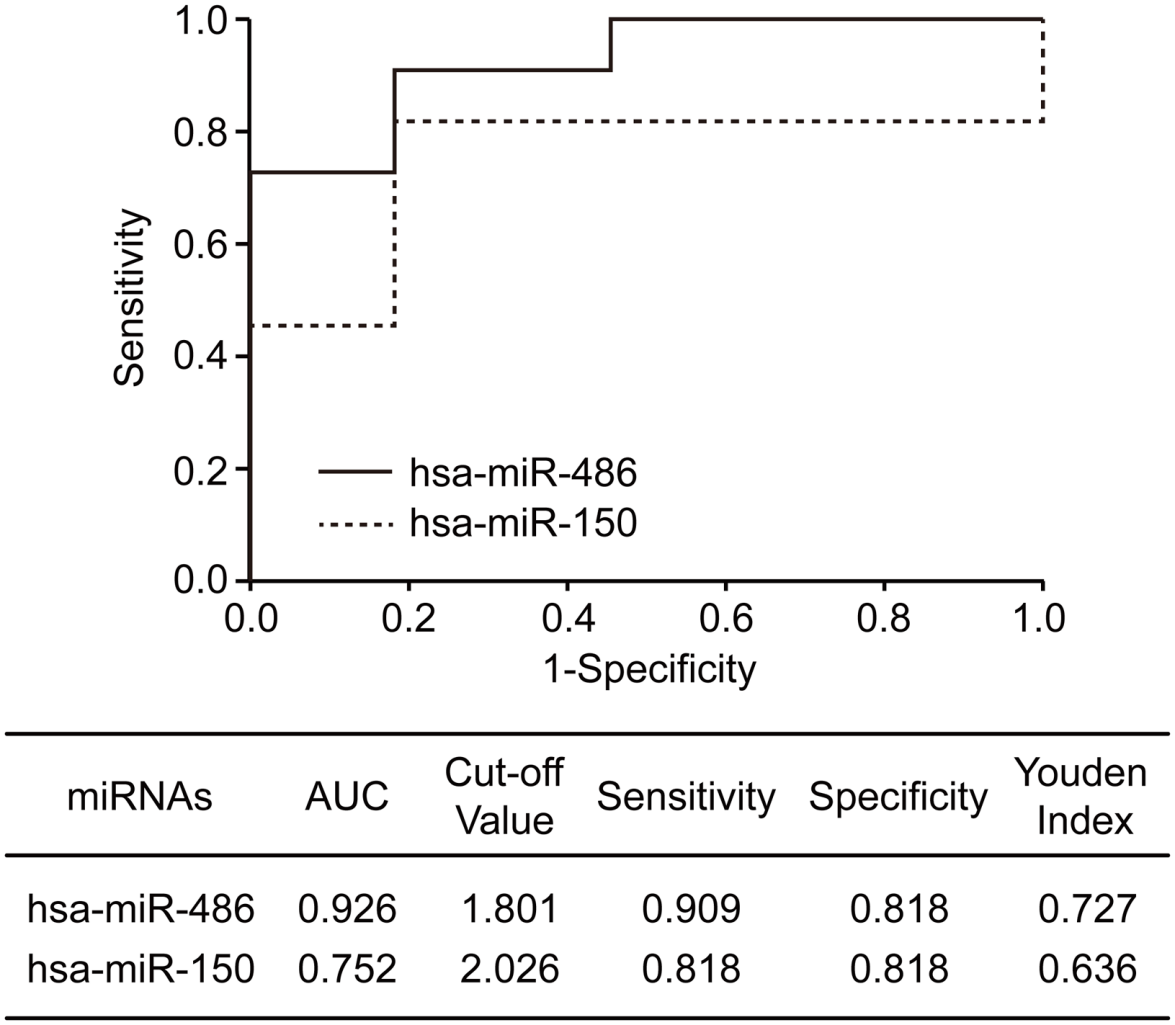
ROC analysis of miR-486 and miR-150. ROC analysis was performed with SPSS 16.0 software. The solid and dotted line indicated ROC curve of miR-486 and miR-150, respectively. AUC, area under curve.

To further explore the clinical utility of miR-486 and miR-150, we performed Receiver Operating Characteristics (ROC) analysis on the plasma miRNA data from lung cancer patients and normal control individuals. The analysis observed that area under curve (AUC), sensitivity and specificity for miR-486 were 0.926, 0.909 and 0.818, respectively, and those for miR-150 were 0.752, 0.818 and 0.818, respectively (Fig 3). The findings implicate differential expressions of miR-486 and miR-150 as potential blood-based biomarkers for detection and diagnosis of lung cancer.

### Down-regulated plasma miR-486 level after surgery is associated with prolonged recurrence-free survival of NSCLC patients

To determine potential roles of miR-486 and miR-150 in predicting clinical outcomes of lung cancer patients, fold changes in plasma levels of miR-486 and miR-150 before and after surgery were recorded and were correlated with survival of the patients. Relative expression levels and fold changes of miR-486 and miR-150 before and after surgery for individual patients are shown in Table 2. All 11 patients were followed after initial surgery and the maximal follow-up period reached 24 months. During the follow-up, three patients (patient No., 101, 103 and 152) were diagnosed as local recurrence or distal metastasis, while the other eight patients were diagnosed as no recurrence Table 2).

Among 11 patients, majority of the patients had a significantly reduced plasma levels after surgery for both miR-486 and miR-150 (Table 2). We divided the patients into two groups according to the changes of miRNA levels after surgery. Patients with significantly reduced miRNA levels in plasma (fold change <0.8) were categorized into down-regulated (DR) group, and the rest patients into unreduced (UR) group. Survival analysis of two groups of patients showed that down-regulated miR-486 level in plasma was associated with prolonged survival of the patients with unreached median survival for DR and 19 months for UR group (Fig 3, hazard ratio, 0.1053; 95% CI, 0.01045 to 1.060; *p* = 0.0561). Although the difference does not reach statistically significant level due to limited number of patients for the study, the trend of association is obvious and the finding supports a further study in a large patient cohort. No difference in patient survival for plasma miR-150 level was observed between DR and UR groups (Fig 3, hazard ratio, 0.7936; 95% CI, 0.07682 to 8.198; *P* = 0.7936).

## Discussion

NSCLC is a leading cause of cancer death worldwide and early diagnosis is essential for prognosis of the patients [1, 2]. Lack of diagnostic biomarkers for early detection has made lung cancer one of the human cancers with the worst prognosis. Circulating miRNAs have been reported to be remarkable bio-markers for cancer diagnosis due to their abundance and stability in circulating blood [6–8]. In this study, we compared plasma levels of ten miRNAs which have been reported to have altered expression in NSCLC [7–16], between healthy volunteers and early stage NSCLC patients. Our study identified two candidate miRNAsmiR-486 and miR-150 as potential blood-based biomarkers for early diagnosis and prognosis of NSCLC patients (Fig 1).

Serum miR-150 has been reported to be aberrantly increased in early stage NSCLC patients and significantly correlated with distant metastasis [14]. MiR-150 promotes proliferation of lung cancer cells by targeting P53 [21, 22] and BAK1 [23], and therefore plays an important mechanistic role in pathogenesis of lung cancer. Studies on diagnostic value of miR-150, especially for early stages of lung cancer, are limited. Consistent with previous reports[21–23], our study observed significantly increased expressions of miR-150 in both cancer tissues and plasma of early stage NSCLC patients. More importantly, we showed that higher level of miR-150 could be detected in plasma of early stages of lung cancer patients and was able to distinguish lung cancer patients from healthy individuals with AUC value of 0.752 (Fig 2). This finding implicates miR-150 as a blood-based candidate biomarker for early detection of lung cancer.

Another interesting finding from current study is that miR-486 appears as a more significant biomarker for clinical assessment of lung cancer patients. Data accumulated from different previous studies on miR-486 in lung cancer were controversial. Peng et al have reported that miR-486 is significantly down-regulated in cancers [24]. Wang et al further reported that down-regulation of miR-486 in lung cancer significantly correlated to stages and lymph metastasis of patients [25]. Study by Solomides et al revealed that miR-486 is reduced in lung cancer tissues [10]. However, a study of miR-486 showed that up-regulated miR-486 level in plasma was correlated with short overall survival [8]. In this study, we demonstrated that miR-486 was upregulated in plasma of the patients with NSCLC and appears as a more sensitive and specific marker in plasma for differentiating early stages of lung cancer from normal individuals(AUC, 0.926, Fig 2), as compared to miR-150. This finding suggests that miR-486 is a more promising marker in peripheral blood for early detection of lung cancer. We realize that our result is inconsistent with the findings from some of the previous studies which reported constant down-regulation of miR-486 in lung cancer[10, 24, 25]. This inconsistence may be due to racial difference in the cohorts for the study.

**Fig 4:**
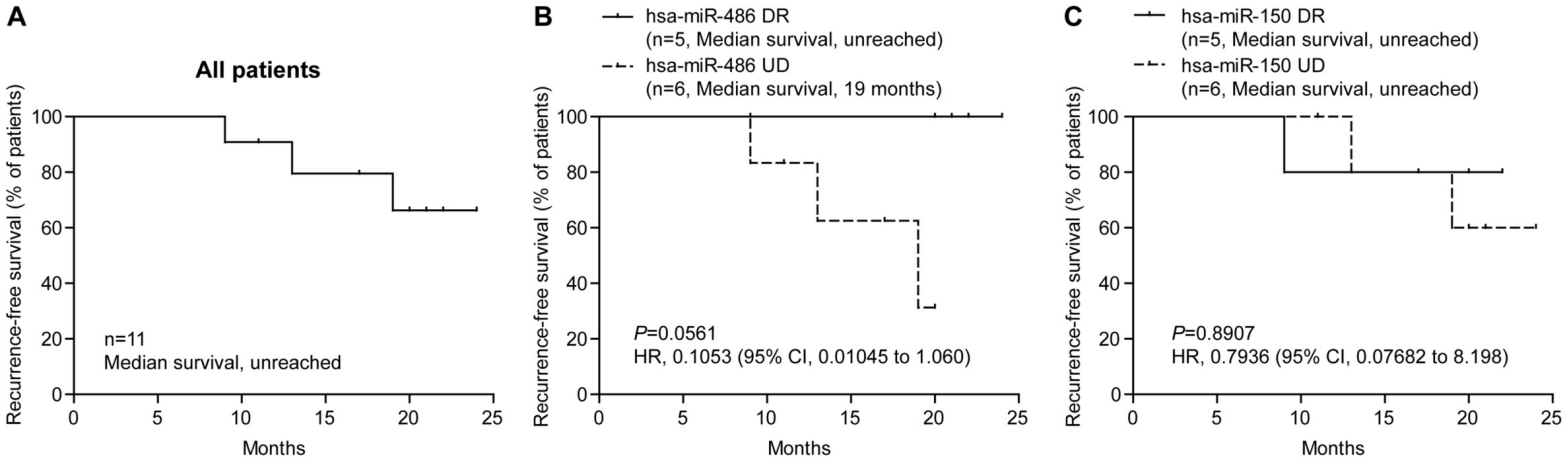
Associations of changes of plasma levels for miR-486 and miR-150 before and after surgery and recurrence-free survival of patients. Association of RFS and plasma miRNA level changes before and after surgery was plotted with Log-rank (Mantel-Cox) Test. (A) Overall RFS of all NSCLC patients. (B) Association of RFS and change of plasma miR-486 level before and after surgery. (C) Association of RFS and change of plasma miR-150 level before and after surgery. DR, down-regulated; UD, un-down-regulated; HR, hazard ratio; CI, confidence interval.

It has been demonstrated that aberrant miRNA levels in peripheral blood of cancer patients are caused by deregulated miRNA expression in tumor tissues which secret tumor-derived miRNAs into body fluids including peripheral blood [15]. To determine if abnormal levels of miR486 and miR150 in plasma are tumor specific, we compared corresponding tissue miRNA levels and plasma miRNA levels. We found that that NSCLC tissue miR-486 levels were significantly correlated with plasma levels, while no statistical correlation was observed in miR-150 levels (Fig 3). These results imply that miR-486 might be more tumor-specific than miR-150. Based on this finding, we hypothesized that levels of miRNAs in plasma can be used for monitoring local status of tumors, for example recurrence after surgery. To test this hypothesis, we compared miR-486 level in plasma before and after surgery. We found that levels of miR-486 in plasma significantly decreased in majority of the patients after surgical removals of tumors while only 3 cases remained high levels of miR-486 in plasma. Interestingly, analysis of association of plasma levels of miR-486 with RFS follow-up data of the patients revealed a tight correlation of changes of plasma miR-486 with recurrent statuses of the patients. Patients with down-regulated plasma miR-486 levels after surgery had prolonged RFS than those with high levels of miR-486 in plasma (Fig 4). This finding implicates that changes of miR-468 levels in plasma in response to surgery can be a promising blood-based marker for predicting local recurrence of tumors after surgery in NSCLC patients. Due to the limited number of patients in this study, a large cohort of patients is under recruitment in our continuing study to further verify this important finding.

## Conclusions

Plasma levels of miR-486 and miR-150 can be potential bio-markers for early diagnosis of NSCLC patients. Monitoring plasma miR-486 levels before and after surgery might predict risk of recurrence for early stage of NSCLC.

## Supporting Information

S1 Table Target sequences of the miRNA probes. (DOCX)
