# Correction: MicroRNA-486 as a Biomarker for Early Diagnosis and Recurrence of Non-Small Cell Lung Cancer

**DOI:** 10.1371/journal.pone.0148589

**Published:** 2016-01-29

**Authors:** Wanshuai Li, Yong Wang, Qi Zhang, Lili Tang, Xiaoping Liu, Yunhua Dai, Liang Xiao, Shuguang Huang, Lu Chen, Zhongmin Guo, Kai Yuan, Jim Lu

The authors are listed out of order. Please view the correct author order and affiliations here: Wanshuai Li^2^, Yong Wang^1^, Qi Zhang^2^, Lili Tang^2^, Xiaoping Liu^2^, Yunhua Dai^2^, Liang Xiao^2^, Shuguang Huang^2^, Lu Chen^2^, Zhongmin Guo^2,3^, Jim Lu^2,3^, Kai Yuan^1^

1 The Department of Cardiothoracic Surgery, No. 2 People's Hospital of Changzhou, 29 Xinglong Xiang, Changzhou, Jiangsu, 213004, China, 2 Changzhou GoPath Diagnostic Laboratory Co. Ltd, 801 Changwuzhong Road, Changzhou, Jiangsu, 213164, China, 3 GoPath Laboratories LLC, 1351 Barclay Blvd, Buffalo Grove, Illinois, 60089, United States of America
