# Supplementary material for: MicroRNA-486 as a Biomarker for Early Diagnosis and Recurrence of Non-Small Cell Lung Cancer
Source: PLoS One. 2015 Aug 3;10(8):e0134220. doi: 10.1371/journal.pone.0134220 (PMC4523212; doi:10.1371/journal.pone.0134220)
Supplement: S1 Table — (DOCX) [file pone.0134220.s001.docx]

**S1 Table Target sequences of the miRNA probes**

| miRNAs | Target sequences (5’-3’) |
| --- | --- |
| hsa-miR-126 | UCGUACCGUGAGUAAUAAUGC |
| hsa-miR-150 | UCUCCCAACCCUUGUACCAGUG |
| hsa-miR-155 | UUAAUGCUAAUCGUGAUAGGGG |
| hsa-miR-205 | UCCUUCAUUCCACCGGAGUCUG |
| hsa-miR-21 | UAGCUUAUCAGACUGAUGUUGA |
| hsa-miR-210 | CUGUGCGUGUGACAGCGGCUGA |
| hsa-miR-26b | UUCAAGUAAUUCAGGAUAGGUU |
| hsa-miR-34a | UGGCAGUGUCUUAGCUGGUUGUU |
| hsa-miR-451 | AAACCGUUACCAUUACUGAGUUU |
| hsa-miR-486 | UCCUGUACUGAGCUGCCCCGAG |
| cel-miR-39 | UCACCGGGUGUAAAUCAGCUUG |
| RNU44 | CCUGGAUGAUGAUAGCAAAUGCUGACUGAA  CAUGAAGGUCUUAAUUAGCUCUAACUGACU |
